# Percutaneous MR guided direct left atrial access to deliver large interventional devices

**DOI:** 10.1186/1532-429X-17-S1-O19

**Published:** 2015-02-03

**Authors:** Toby Rogers, William Schenke, Jonathan R Mazal, Merdim Sonmez, Ozgur Kocaturk, Kanishka Ratnayaka, Michael Hansen, Anthony Z Faranesh, Robert J Lederman

**Affiliations:** 1 National Heart Lung and Blood Institute, National Institues of Health, Bethesda, MD, USA; 2 Institute of Biomedical Engineering, Bogazici University, Istanbul, Turkey; 3 Department of Cardiology, Children's National Medical Center, Washington, DC, USA

## Background

**Figure 1:**
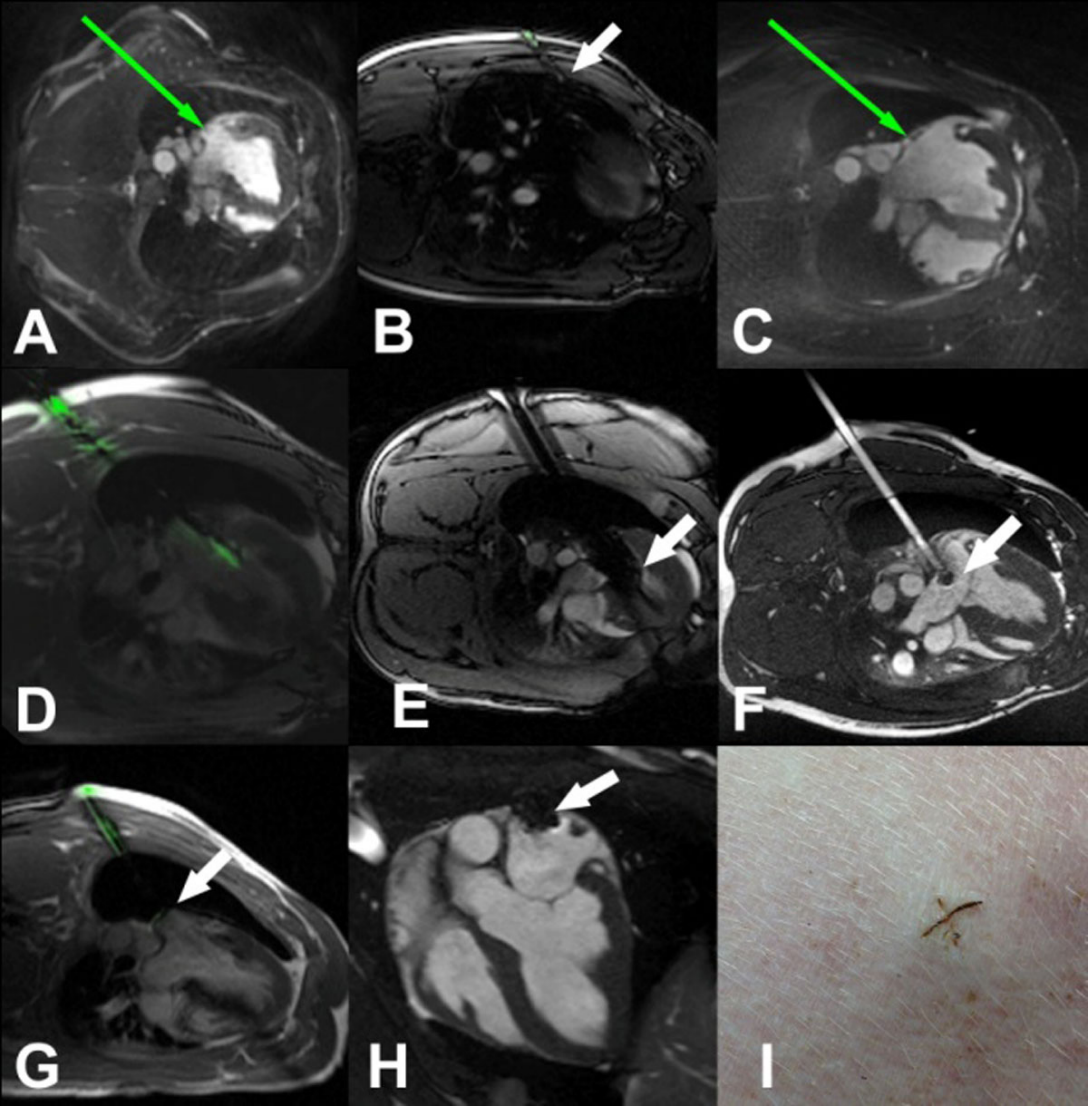


Transcatheter aortic valves have benefitted from device miniaturization and in-situ assembly to reduce delivery system caliber and enable trans-vascular delivery. In contrast, investigational transcatheter mitral prostheses are bulky devices that require large caliber access ports. Trans-apical delivery is undesirable because of increased morbidity associated with rib spreading, closure site bleeding and impact on left ventricle (LV) function. Trans-septal delivery is challenging because of the acute angle required to reach the mitral valve. A ‘straight shot' to the mitral valve (Fig 1A) that does not violate the LV myocardium is preferable, both in terms of device engineering and patient outcome. We hypothesized that with realtime MR guidance and by deflating a lung, it is possible to access the left atrium (LA) directly through the posterior chest wall, and close the access port using off-the-shelf nitinol devices.

## Methods

LA access was obtained in 8 naïve Yorkshire swine. Animals were positioned on their right side. An active MR needle was used to access the left pleural space and insert a drain (arrow, Fig 1B) to insufflate the pleural space with CO_2_. A direct trajectory to the LA was planned (Fig 1C) and the active MR needle was used to puncture through the chest wall, passing through the empty pleural space and enter the LA posteriorly (Fig 1D). Position was confirmed by pressure waveform and by injecting gadolinium. A stiff wire was introduced to the LV apex, over which an 18Fr sheath with a passive MR marker at the tip was advanced into the LA (arrow, Fig 1E-F). Sheath position and relation to the mitral valve were assessed using 3D and cine MRI. The sheath was withdrawn and the LA puncture was closed with a nitinol closure device (arrow, Fig 1G). Animals were re-imaged 7days later.

## Results

Pleural access and left lung deflation was uncomplicated in all 8 animals. Realtime MR guided LA access was successful in all (with a single pass in 7/8, but required a second pass because of rudimentary device failure in 1/8). 3D and cine MRI confirmed that the 18Fr sheath trajectory relative to the plane of the mitral valve was favorable to perform a mitral intervention (Fig 1F). The LA puncture was successfully closed with nitinol closure devices in all animals (under X-ray guidance in 6/8 and under MR guidance in 2/8). The lung was re-inflated by aspirating the pleural CO_2_. There were no significant peri-procedural complications or mortality. After 7days, MRI confirmed stable position of the LA closure device (arrow, Fig 1H). Only one significant pericardial effusion was observed in the one animal in which the LA was punctured twice.

## Conclusions

Percutaneous (Fig 1I) MR guided direct LA access with large sheaths is feasible in swine to achieve a straight trajectory to the mitral valve without injuring the LV myocardium. The puncture is closed using nitinol closure devices. This technique could provide a simpler and safer access route for transcatheter mitral valve interventions.

## Funding

This work was supported by the Division of Intramural Research, National Heart Lung and Blood Institute, National Institutes of Health (Z01-HL005062).

